# Non-Coding RNAs in the Brain-Heart Axis: The Case of Parkinson’s Disease

**DOI:** 10.3390/ijms21186513

**Published:** 2020-09-06

**Authors:** Shubhra Acharya, Antonio Salgado-Somoza, Francesca Maria Stefanizzi, Andrew I. Lumley, Lu Zhang, Enrico Glaab, Patrick May, Yvan Devaux

**Affiliations:** 1 Cardiovascular Research Unit, Department of Population Health, Luxembourg Institute of Health, L-1445 Strassen, Luxembourg; Shubhra.Acharya@lih.lu (S.A.); Antonio.SalgadoSomoza@lih.lu (A.S.-S.); FrancescaMaria.Stefanizzi@lih.lu (F.M.S.); andrew.lumley@lih.lu (A.I.L.); lu.zhang@lih.lu (L.Z.); 2 Faculty of Science, Technology and Medicine, University of Luxembourg, L-4365 Esch-sur-Alzette, Luxembourg; 3 Luxembourg Centre for Systems Biomedicine, University of Luxembourg, L-4365 Esch-sur-Alzette, Luxembourg; enrico.glaab@uni.lu (E.G.); patrick.may@uni.lu (P.M.)

**Keywords:** Parkinson’s disease, brain, heart, biomarkers, non-coding RNAs, data science, systems biomedicine, artificial intelligence

## Abstract

Parkinson’s disease (PD) is a complex and heterogeneous disorder involving multiple genetic and environmental influences. Although a wide range of PD risk factors and clinical markers for the symptomatic motor stage of the disease have been identified, there are still no reliable biomarkers available for the early pre-motor phase of PD and for predicting disease progression. High-throughput RNA-based biomarker profiling and modeling may provide a means to exploit the joint information content from a multitude of markers to derive diagnostic and prognostic signatures. In the field of PD biomarker research, currently, no clinically validated RNA-based biomarker models are available, but previous studies reported several significantly disease-associated changes in RNA abundances and activities in multiple human tissues and body fluids. Here, we review the current knowledge of the regulation and function of non-coding RNAs in PD, focusing on microRNAs, long non-coding RNAs, and circular RNAs. Since there is growing evidence for functional interactions between the heart and the brain, we discuss the benefits of studying the role of non-coding RNAs in organ interactions when deciphering the complex regulatory networks involved in PD progression. We finally review important concepts of harmonization and curation of high throughput datasets, and we discuss the potential of systems biomedicine to derive and evaluate RNA biomarker signatures from high-throughput expression data.

## 1. Introduction

Parkinsonian disorders are considered the second most common neurological diseases in humans [1]. Currently, more than 1.2 million people across Europe have Parkinson’s disease (PD). Taking into account the aging of the population due to the extension of life expectancy, it is predicted that the prevalence of PD will almost double in the next 30 years [2].

The most recognizable symptom of the disease is the presence of tremors, although the disease could be manifested with bradykinesia and other non-motor symptoms such as the accumulation of an aberrant form of alpha-synuclein or the mitochondrial dysfunction [3]. This symptomatology arises after the loss of dopaminergic neurons in specific areas of the brain, like the motor cortex, the substantia nigra, and the thalamus. The underlying pathology denotes neurodegeneration in specific areas of the brain that evokes the tremors, which are responsible for several degrees of disability of the patients. These areas are mainly occupied by dopaminergic neurons, with PD patients presenting defects in the production of the essential neurotransmitter produced by this cell subpopulation, dopamine. Thus, many of the treatments for PD involve the restoration of dopamine levels in the brain, either with the addition of an upstream metabolite or affecting the recycling of dopamine (increasing its availability in the affected neurons) [4].

L-Dopa is a treatment of choice for most PD patients, although it has deleterious side effects in the cardiovascular system, such as aortic stiffness and diastolic function [5]. Some PD patients show other cardiac abnormalities which can lead to sudden cardiac death [6]. Moreover, the complexity of the disease is exacerbated by an important inflammatory component [7,8,9,10,11].

Despite the significant progress in the understanding of the mechanisms leading to PD, the clinical characterization of patients remains challenging [12]. The main hurdle to be overcome is the complexity of the phenotypes, combining several motor and non-motor symptoms and ranging from the early onset of the disease to the advanced stages. Parkinsonian disorders englobe not only PD but also Lewy body disease and other atypical parkinsonian syndromes that represent a challenge for differential diagnosis in the earlier stages [13,14,15]. In addition, the classification of advanced stages of PD involves a complicated combination of clinical, behavioral, and motor parameters into a single scale recommended by the International Parkinson and Movement Disorder Society (MDS-UPDRS scale) to determine the extent of the disease progression [16]. PD is diagnosed through clinical tests, including extensive neurological and physical examination [17], and post-mortem neurological examination is necessary for disease confirmation [18]. Being able to identify novel methods to accurately diagnose and risk-stratify PD patients may help to move towards personalized healthcare. New biomarkers may help both to diagnose, risk stratify, and prognosticate PD patients at an early stage of the disease.

Due to their presence and relative stability in the bloodstream—often packed in extracellular vesicles or exosomes—non-coding RNAs appeared as potential disease markers [19]. Since non-coding RNAs affect multiple biological pathways leading to disease development and progression, they are also promising therapeutic targets [20]. Several studies reported a permeability of the brain-blood barrier in PD patients [21,22,23,24], which suggests that non-coding RNAs released from the brain into the blood may serve as biomarkers in these patients. Non-coding RNAs are arbitrarily classified in two groups depending on their length. The widely investigated microRNAs (miRNAs) are 20–22 nucleotides long, small RNA molecules known to downregulate the expression on protein-coding RNAs (messenger RNAs). Long non-coding RNAs (lncRNAs) usually display a nucleotide sequence of more than 200 nucleotides, and regulate gene expression through diverse mechanisms, mostly at the epigenetic level [25]. LncRNAs are present in the cells and in the extracellular compartment under either a linear or a circular form. In the latter case, they are named circular RNAs (circRNAs).

This review article presents an overview of the knowledge of the role of non-coding RNAs in PD, focusing on the interactions between the brain and the heart.

## 2. MicroRNAs

### 2.1. Regulation of miRNAs in Parkinson’s Disease

Table 1 gathers the current knowledge of the regulation of miRNAs in brain and body fluid samples of PD patients. In the exploration of new biomarkers of neurodegenerative diseases in body fluids, the logic drives the search to the cerebrospinal fluid (CSF) due to its proximity with neurons. Moreover, the study of CSF overcomes the issue of potential low permeability of the blood-brain barrier, hampering the release of indicators of brain damage into the bloodstream. In this regard, CSF levels of miR-205 and miR-24 were different between PD patients and non-neurological controls [26]. However, in another study, miR-24 expression levels in CSF were stable between PD patients and controls and was even used as the internal control in quantitative RT-PCR experiments [27]. In this study, three miRNAs were upregulated in PD patients as compared to controls: miR144-5p, miR-200a-3p, and miR-542-5p [27]. In yet another study, miRNA profiles in exosomes of CSF samples from PD patients and controls were generated using microarrays. Several miRNAs showed an upregulation (miR-153, miR-409-3p, miR-10a-5p, let-7g3p, miR-136-3p, and miR-433), others were downregulated (miR-1 and miR-19b-3p) in PD patients compared to controls [28]. Using a PCR array, miR-4274 was identified as a candidate marker for the diagnosis of PD versus idiopathic normal pressure hydrocephalus (a condition with similar symptomatology) [29]. Unfortunately, these studies included less than 50 patients or controls per group, and are therefore underpowered. The results have to be considered with caution and need independent validation in adequately sized cohorts. Nevertheless, these studies have the merit of having uncovered the regulation of miRNA expression levels in CSF of PD patients. The difficulty of obtaining CSF hampers the clinical applicability of these miRNAs.

The biological fluid of choice to search for miRNA biomarkers is undoubtedly the peripheral blood. Interestingly, a study using both serum and CSF samples from post-mortem PD patients and neurologically normal controls reported the regulation of different miRNAs in body fluids, some of them correlated with disease severity as assessed by Braak stages or by the presence of Lewy bodies [58]. These observations suggest that blood miRNAs may constitute a pool of easily accessible markers of neurological damage in PD patients, as previously described for the neurological outcome of patients with cardiac arrest [59,60]. Exosome fractions of serum samples from PD patients and controls were used to address the regulation of 24 miRNAs previously linked to PD [31]. Among these, miR-24 and miR-195 were upregulated, whilst miR-19b was downregulated in PD patients compared to controls [31]. The upregulation of serum levels of miR-24 in this study contrasts with the downregulation observed in the CSF of PD patients from another study [26], supporting the hypothesis that miR-24 might be released from the brain compartment to the blood upon neurological damage associated with PD development. However, as mentioned earlier, CSF levels of miR-24 were found to be stable between PD patients and controls [27], re-emphasizing the need for properly sized patient cohorts and well-defined experimental protocols to draw meaningful conclusions.

Plasma has also been extensively used to search for miRNA biomarkers, although platelets can greatly influence the pool of miRNAs in plasma samples [61]. Using microarrays covering 866 miRNAs, miR-222, miR-505, were found to be differentially expressed and presented an interesting biomarker value for PD [39] (Table 1). A PCR-based study with plasma isolated from 31 PD and 25 controls revealed that miR-331-5p was upregulated in PD patients [40]. In other small-scale studies, miR-124 was downregulated, and miR-137 and miR-30a-5p, were upregulated in plasma samples of PD patients compared to controls [41,42]. In a recent study with 319 idiopathic PD and 273 control samples, plasma levels of miR-105-5p were upregulated in PD samples [43].

Peripheral blood cells contain large amounts of miRNAs and, since their composition in the blood varies depending on the inflammatory status, they constitute another reservoir of potential disease biomarkers [62]. PD is associated with systemic inflammation hence the capacity of the blood cell transcriptome to reveal miRNAs with diagnostic or prognostic value. A microarray study profiled 763 miRNAs in peripheral blood mononuclear cells (PBMC) from 19 PD patients and 13 controls [52]. Four hundred ninety miRNAs were detected, 18 were downregulated while none was significantly upregulated [52]. Expression levels of miR-29c, miR-424, miR-30e5p, miR-103a-3p, miR-30b-5p, miR-29a-3p, and miR-155-5p were upregulated in PBMC isolated from PD subjects as compared to control PBMCs [49,50,51], while miR-146a-5p was downregulated [51]. A Taqman PCR-based study in RNA samples isolated from peripheral lymphocytes of 20 non-medicated PD patients and 18 PD patients under medication revealed that several miRNAs, including brain-enriched miRNAs, were upregulated after treatment (miR-7, miR9-5p, miR-9-3p, miR-129, and miR-132) whilst other miRNAs were unaffected (miR-133b, miR-153, miR-191, miR-346, miR-433, miR-598) [55]. A large scale study used whole blood samples from two different cohorts—PPMI (2802 PD vs. 1538 controls) and NCER-PD (947 PD vs. 493 controls) to highlight the de-regulated small ncRNAs, in particular miRNAs, in PD and demonstrated that the occurrence of their de-regulation is related to disease progression and aging [63]. Whether these changes merely reflect the control of inflammation or the amelioration of the neurological status of the patients remains to be determined.

Recent studies have also shown that saliva can be used as a non-invasive source to detect the change in expression of circulatory miRNAs in patients with PD as compared to controls. Using saliva samples from 83 PD patients and 77 non-neurological controls, Cressatti et al. showed that miR-153 and miR-223 expression levels were significantly downregulated in PD vs. controls [56]. Another RT-qPCR based study on saliva samples from 30 PD patients vs. 30 healthy controls revealed the increased expression of miR-874 and miR-145-3p in PD patients as compared to controls. Additionally, these miRNAs were predicted to target the protein-coding gene DJ1 (PARK7) and are thought to help in the protection against oxidative stress [57]. These studies show that circulatory miRNAs found in various body fluids, such as saliva may act as promising biomarkers for early detection and prognosis of PD.

### 2.2. Functional Roles of miRNAs in Parkinson’s Disease

**Figure 1:**
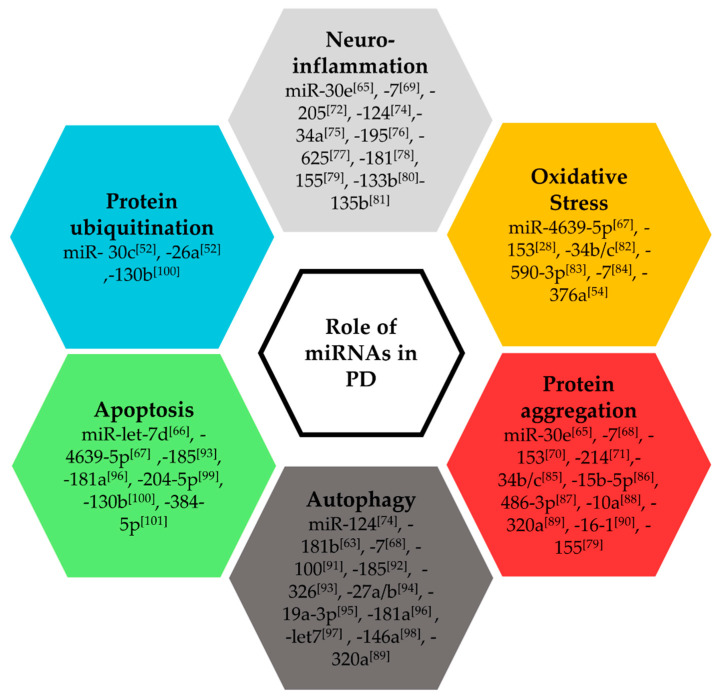
Role of microRNAs in Parkinson’s disease [28,52,54,63,65,66,67,68,69,70,71,72,74,75,76,77,78,79,80,81,82,83,84,85,86,87,88,89,90,91,92,93,94,95,96,97,98,99,100,101].

Several investigations were conducted to gain insights into the functional role of miRNAs in PD. MicroRNAs have been shown to regulate a substantial number of mechanisms involved in PD progression (Figure 1). The miR-30 family, whose expression is regulated in CSF and post-mortem brain samples of PD patients [28,64], plays a protective role against neuroinflammation. Indeed, in a PD mouse model, miR-30e reversed the loss of tyrosine hydroxylase (TH), α-synuclein aggregation, and motor symptoms induced by MPTP treatment [65]. A study in dopaminergic MN9D cells treated with 6-hydroxydopamine (6-OHDA) showed that increased expression of let-7d reduces cell apoptosis [66]. In another study, using human embryonic kidney epithelial cells (HEK293T) and neuronal SH-SY5Y cells, miR-4639-5p targeted the protein deglycase DJ-1 (also known as PARK7, a PD-related gene), causing oxidative stress and neuronal apoptosis [67]. MiR-7 reduced α-synuclein aggregation in human neural progenitor cells [68] and protected against the loss of dopaminergic neurons in MPTP-treated mice [69]. MiR-153 helped in the suppression of α-synuclein via mitochondrial reactive oxygen species regulation in HEK293T cells treated with MPP [70]. In a PD mouse model and SH-SY5Y cells treated with MPP, the loss of miR-214 was associated with increased expression of α-synuclein [71]. In a miRNA expression profiling study on 19 PD patients and 13 controls, miR-30c and miR-26a were predicted to be the main modulators of the protein ubiquitination pathway [52]. In a study combining investigations in PD patient samples and validation in cell models, miR-205 regulated the expression of PD-related leucine-rich repeat kinase 2 (LRRK2), and prevented the defects in neurite outgrowth in LRRK2 mutant neurons [72]. MiR-181b and miR-124 were shown to be involved in inflammatory response and autophagy in PD models [73,74]. Together, these studies support a significant regulation of PD pathogenesis by miRNAs.

### 2.3. Therapeutic Potential of miRNAs in Parkinson’s Disease

In addition to their potential as PD biomarkers, miRNAs also have some promise as therapeutic targets. A first approach was explored with a miR-30 shRNA-like molecule, which was able to target LRRK2 [102]. Another miR-based strategy was the creation of artificial mirtrons to target PD-linked LRRK2 and α-synuclein messenger RNAs using as a basis the mirtron miR-1224 [103]. An adenovirus-associated vector with a shRNA sequence embedded into a miRNA sequence was able to silence alpha-synuclein in vitro and showed lower toxicity than a similar shRNA under an H1 promoter only [104]. The human cytomegalovirus non-coding Beta2.7 RNA has been proposed as a novel potential therapeutic approach for PD [105]. Another therapeutic approach was carried out in a 6-hydroxydopamine mouse model for PD using nanoparticles coated with miR-124, which ameliorate the PD phenotype at the cellular and motor level [106]. Using the same model together with studies in SH-SY5Y and PC12 cell lines, others reported that the neuroprotective effect of miR-124-3p could be partially due to a decrease of the ANXA5/ERK signaling pathway (which leads to apoptosis) [107]. Therefore, several therapeutic approaches based on miRNAs show promising results that need to be confirmed.

## 3. Long Non-Coding RNAs

### 3.1. Regulation of lncRNAs in Parkinson’ Disease

The expression levels of lncRNAs are regulated during normal brain development, as described elsewhere [108,109]. Moreover, there is increasing evidence for the involvement of lncRNAs in the onset and progression of neurodegenerative disorders [110,111]. While lncRNAs are canonically expressed under a linear form, a back-splicing event between two or more exons can lead to circularization of the linear transcript, generating a circular RNA [112]. To date, relatively few studies have addressed the biomarker value of lncRNAs and circRNAs, even though their biomarker potential has been reported in other diseases, such as cardiovascular disease [113]. The identification of single nucleotide polymorphisms in four lncRNAs in patients with PD suggests that genetic variations within lncRNAs, as well as lncRNAs themselves, may be involved in PD development [114].

There is growing evidence that lncRNAs are regulated in PD (Table 2). In a study using human brain specimens from 20 PD patients and 10 controls, the lncRNA H19 was downregulated whilst lincRNA-p21, MALAT1, SNHG1, and NEAT1 were upregulated in PD [115]. Subsequent studies validated the up-regulation of NEAT1 in the substantia nigra [116] and peripheral blood [117] of PD patients. Another study comparing Substantia nigra samples from PD patients and controls showed a regulation of several lncRNAs, among which AL049437 (deleterious) and AK021630 (protective) were the most regulated ones in a knock-down-based cellular model [118]. In a study using RNA sequencing in leukocytes from PD patients and age- and sex-matched healthy controls, two lncRNAs named U1 and RP11-462G22.1 were regulated, and subsequently validated by PCR [119]. Elsewhere, the differential expression of RP11-462G22.1 could be confirmed when comparing cerebrospinal fluid exosomes from PD patients and healthy controls [28]. Finally, elevated levels of lncRNA NEAT1 have been described in the peripheral blood of PD patients [117].

### 3.2. Functional Roles of lncRNAs in Parkinson’s Disease

**Figure 2:**
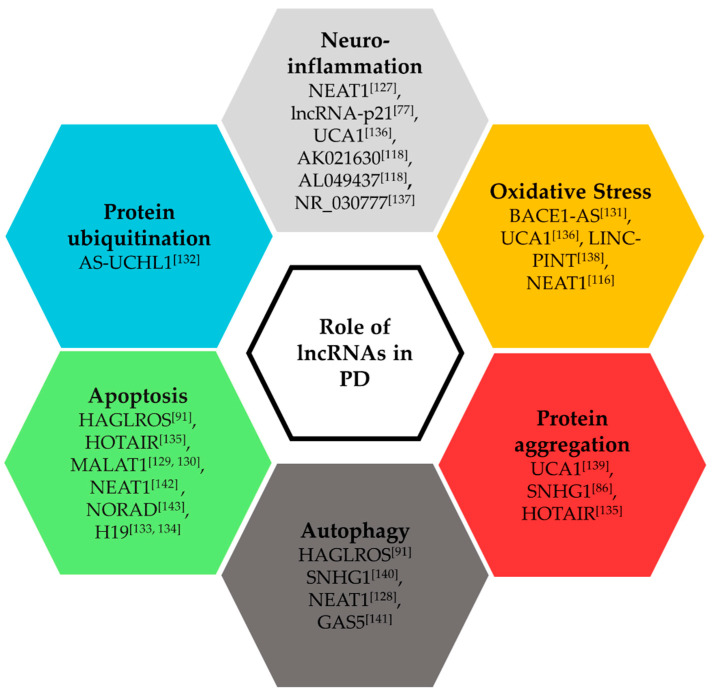
Role of lncRNAs in Parkinson’s disease [77,86,91,116,118,127,128,129,130,131,132,135,136,137,138,139,140,141,142,143].

The development of PD involves the disturbance of several biological processes important for neuronal survival. Midbrain dopaminergic neurons are the most affected in PD. Thus, the development of neurons and regeneration post neuronal injury plays an important role in the PD processes. Several studies in different model systems have illustrated the roles of lncRNAs in these biological processes, and thus, during the development of PD (Figure 2). Experimental studies showed that the lncRNAs SNGH1 and AL049437 contribute to MPP cytotoxicity in neuronal SH-SY5Y cells [80,122,123]. The lncRNA HOTAIR promotes the PD phenotype induced by MPTP in mice and MPP in SH-SY5Y cells by upregulating the leucine-rich repeat kinase 2 LRRK2, an enzyme involved in PD development [124]. The lncRNA microtubule-associated protein tau antisense 1 (MAPT-AS1) was decreased in different regions of the brain of PD patients as compared to healthy matched controls, and has been proposed as an epigenetic regulator of MAPT expression, which has a pathogenic role in PD [125]. Overexpression of a green fluorescent protein-coupled α-synuclein in cells revealed differences in the expression levels of lncRNAs [126]. NEAT1 expression was significantly upregulated in SH-SY5Y cells treated with MPP, in a PD mouse model and in the substantia nigra of PD patients, and is involved in the protection against oxidative stress and neuronal injury, and promote autophagy [116,127,128]. LncRNA-p21 was shown to regulate the MPP-induced neuronal injury in SH-SY5Y cells via the miR-626-TRMP2 regulatory network [77]. MALAT1 acts as a mediator of cell apoptosis in PD mice and cellular models [129,130]. Further, the downregulation of lncRNA BACE1-AS in a PD rat model was reported to reduce the production of nitric oxide synthase, and thus, prevent oxidative stress [131]. LncRNA HAGLROS is highly expressed in a PD mouse model, as well as in SH-SY5Y cells and is associated with the inhibition of apoptosis and autophagy [91].

Research has highlighted protein ubiquitination as another important process that is disturbed in PD. LncRNA AS-UCHL1, an antisense transcript of the UCHL1 gene, induces UCHL1 translation, thereby showing a protective effect against PD as seen in in vitro and in vivo models [132]. Recent studies have revealed that lncRNA H19, which has previously been studied in different cancers and heart diseases, can also play a protective role against dopaminergic neuronal loss and apoptosis via regulating miR-301b-3p and miR-585-3p, respectively, in PD mice models [133,134].

As extensively studied and illustrated before, α-synuclein protein aggregation is a prominent molecular characteristic of PD. A study in a PD mouse model showed that when the lncRNA HOTAIR was knocked-down, this led to a reduction in the number of α-synuclein positive cells, and thus, apoptosis of dopaminergic neurons [135]. Taken together, the above studies suggest that there are various factors that work together in the progression of the disease; therefore, having in-depth knowledge about the role of lncRNAs in these processes could give better insights about the early diagnostic and effective therapeutic measures.

## 4. Circular RNAs

Circular RNAs are differentialy expressed in various brain and subcellular compartments where they accumulate with age [144]. Since age is a risk factor for neurodegeneration in PD, it is intuitive to think that circRNAs might contribute to the pathogenesis of PD. Dysregulation of gene expression and splicing, affecting, for instance, the expression profiles of circRNAs, often accompanies brain damage. The highly expressed circRNA CDR1-AS appears to be downregulated in the hippocampal CA1 region of patients with sporadic Alzheimer’s disease [145]. Due to its high capacity to bind miR-7, which has a protective role in neuronal death [146], it is tempting to speculate that the CDR1-AS-miR-7 axis may also play a role in PD. Another study based on miRNA/SNCA regulation on an MPP treated SH-SY5Y cell model that aimed to delineate the mechanism of pramipexole administration in PD patients showed a downregulation of circSNCA and SNCA expression post pramipexole treatment. The circSNCA downregulation could upregulate miR-7, thereby downregulating the expression of SNCA and eliciting a reduction of cell apoptosis and increased autophagy [147]. A recent study in both mouse and cell models showed circDLGAP4 to be downregulated in both models of PD and that this circRNA could exert neuroprotective effects by regulating miR-134-5p, adding to the importance of circRNA-miRNA interactions in PD pathogenesis [148].

A novel circRNA molecule generated from the zip-2 gene, named circzip-2, was found in a transgenic *C. elegans* model of PD and was shown to be involved in α-synuclein protein aggregation and extended lifespan [149]. Further, a circRNA transcriptome profiling study from different brain regions of MPTP-induced PD mouse model revealed differential expression of six circRNAs predicted to be involved in the PD-related pathways. Through an in silico analysis, mmu-circRNA-0003292, and mmu_circRNA_0001320 were predicted to interact with well-known PD-related miRNAs, miR-132, and miR-124, respectively [150]. A recent study on RNA sequencing of brain samples from PD patients and controls also showed the presence of differentially expressed circRNAs in different brain regions. The expression of circSLC8A1 increased in the substantia nigra of PD brain samples and in a PD cell model [151]. Taken together, these studies provide insights into the possible functional role of circRNAs in PD development.

## 5. tRNA Derived ncRNAs

t-RNA fragments are a distinct class of small ncRNAs derived from processing of mature t-RNAs in the cytoplasm. These can be (a) t-RNA halves, also known as tiRNAs (30–50 nucleotides long), which are stress-induced and are cleaved from mature t-RNA by the ribonuclease angiogenin (ANG), and (b) tRNA fragments (tRFs) that are 12 to 30 nucleotides long and cleaved from mature tRNA by the endoribonuclease Dicer. These have been found to be dysregulated in different cancers [152,153] and neurological disorders [154]. A study on 3146 PD patients and 7668 controls demonstrated a high prevalence of ANG variants in PD and amyotrophic lateral sclerosis (ALS), showing a possible link between the tRNA processing enzyme –ANG with PD and ALS [155]. A recent analysis of three different data sets (29 patient and 33 control samples from the pre-frontal cortex, CSF samples from 63 patients, and 64 controls, and serum samples from 34 patients and 31 control subjects) revealed that tRFs are differentially abundant in PD vs. controls and were shared among the three different sample types [156]. This study shows that tRFs are detectable in CSF and serum samples and could serve as a potential circulatory biomarker in PD research.

## 6. Cardiac Comorbidities in PD

The development of cardiovascular impairment and loss of cognitive function are major interrelated hallmarks of aging [157,158]. PD and cardiovascular diseases (CVD) share common health (diabetes, hypertension, and obesity [159,160]) and mechanistic (oxidative stress and chronic inflammation [161,162]) risk factors, which supports the need to study these two diseases in concurrence. In this regard, various studies have shown the development of CVDs, such as stroke and acute myocardial infarction in patients with PD [163,164]. A large-scale study with 1948 PD patients subdivided into three different age groups showed 36.3% of patients had circulatory comorbidities, where the prevalence of developing circulation problems was shown to be directly related with age [165]. Another study, including 3367 PD and 823 Parkinsonism patients, showed 42.5% of sufferers had cerebrovascular disease, and 33.2% had hypertension co-occurring with PD, where patients with Parkinsonism exhibited higher chances of suffering from comorbidities, which further increased with age [166]. Due to these comorbidities, it is important to carefully select therapeutic approaches for these patients. The development of cardiac valvulopathy in patients with PD undergoing dopamine agonist therapy (drugs-Pergolide and Cabergoline) has been observed to be most common [167,168]. The detrimental effects of Levodopa—the most common drug used to treat PD—on the heart has been studied for years [169]. PD patients showed reduced mean arterial pressure (15%), cardiac stroke volume (13%), and cardiac contractility (18%) after Levodopa administration [170]. Under L-Dopa treatment, patients had aortic stiffness and impaired diastolic function [5]. Administration of the dopamine agonist Pramipexole increased the risk of heart failure [171]. Conversely, Amiodarone, an antiarrhythmic medication, has been shown to have neurotoxic effects associated with tremors and Parkinsonism [172,173]. Cinnarizine and Flunarizine—calcium channel blocking drugs frequently used to treat angina, high blood pressure, and arrhythmia—induced movement disorders, Parkinsonism, and depression in 34% of patients [174]. Interestingly, PD-associated mitochondrial proteins Parkin, PINK1, DJ-1, LRRK2, and α-synuclein (involved in governing neuro-inflammation) are also expressed in the heart [175], yet their potential functions in the heart have not been deeply studied, especially in human. An experimental study reported that Parkin plays a role in cardiac function and left ventricular remodeling in rats subjected to myocardial infarction [176]. Taken together, these observations support the importance of brain-heart interactions in PD and highlight the need for further research in this area to uncover the potential mechanisms.

## 7. Non-Coding RNAs in the Brain-Heart Axis in PD

One angle to investigate the brain-heart interactions in PD is to focus on non-coding RNAs known to be perturbed in either PD or heart diseases. A study with 46 PD subjects showed a significant reduction in circulating levels of miR-133b [44]. MiR-133b is expressed in the brain and is known to regulate the development of midbrain dopaminergic neurons [177]. Interestingly, miR-133b is also regulated after myocardial infarction and dampens reactive oxygen species production [178,179]. Therefore, miR-133b has roles both in the brain and the heart. Another example is miR-124, along with miR-124-5p and miR-124-3p, which are known for their protective roles in cardiac injury, angiogenesis, and other heart diseases [180,181]. In addition, miR-124-3p is an efficient prognostication tool for neurological outcome and survival in patients post-cardiac arrest [59]. It has antioxidant properties, protects against neuroinflammation, and regulates several pathways involved in PD progression [71,74,107,182,183]. Hence miR-124-3p establishes functional links between brain and heart diseases. Not only small ncRNAs but also long ncRNAs appear to be involved in brain-heart interactions. Indeed, a recent study showed that the lncRNA MALAT1 is a positive regulator of apoptosis and acts by inhibiting miR-124 levels in SH-SY5Y neuronal cell line and mice PD model [130]. MALAT1 is also known as a positive regulator of cardiomyocyte apoptosis and cardiac fibrosis [184,185]. Furthermore, MALAT1 upregulates α-synuclein protein expression in an MPTP-induced PD mouse model and in SH-SY5Y neuronal cells treated with MPP+ [186]. Lastly, MALAT1 promotes apoptosis of MN9D dopaminergic neuronal cells through a mechanism implicating LRRK2 and miR-205-5p [187]. Thus, MALAT1 appears to be a potential target against apoptosis in both heart disease and PD. HOTAIR is another lncRNA known to have prominent roles in PD and heart diseases. It is highly expressed in cardiac tissues and is regulated in plasma and serum samples of patients with congenital heart diseases and acute myocardial infarction [188,189]. HOTAIR supports the stability of LRRK2 expression in PD, and its knockdown in MPP-treated SH-SY5Y cells conferred a protective effect against neuroinflammation [190]. HOTAIR promotes cell apoptosis by sponging miR-221 in in vitro and in vivo PD models [191]. These studies provide insights into the interplay between the diseased heart and brain and suggest that non-coding RNAs play a role in common pathways governing PD and heart diseases.

## 8. Translatable Techniques for Parkinson’s Disease

Through ‘bed to bench to bed’ work cycles, the close partnership between clinics and research laboratories can aid in the uncovering of novel, sensitive, and reproducible biomarkers. Indeed, the use of many laboratory techniques has expanded our knowledge in recent years and continue to play pivotal roles in Parkinson’s clinics. Such techniques as transcriptomics, proteomics, and metabolomics have been able to provide great insight into disease pathogenesis. The applicability of the omics techniques to multiple biological media such as brain tissue, CSF, and blood has allowed uncovering several key processes decisive to PD development—notably related to mitochondrial dysfunction and synaptogenesis [192,193]. Moreover, genome-wide association studies have identified dozens of risk loci for PD, with a recent study uncovering a further 17 [114,194]. The knock-on effects of these discoveries not only have enhanced our understanding of disease pathogenesis but also have been able to suggest novel drug targets. Whilst such studies offer much promise for the future, the use of cutting-edge technology such as CRISPR Cas9 gene editing or induced pluripotent stem cells (iPSCs) have the potential to change the PD landscape today. As an example, iPSCs may be used not only to improve the diagnosis of PD but also to treat this disorder through genetic reprogramming [195].

## 9. Available Gene Expression Datasets in Parkinson’s Disease

A non-systematic search of gene expression omnibus (GEO) database [196] for public datasets associated with Parkinson’s disease identified 53 gene expression datasets generated from human samples, including whole blood, blood cells, iPSCs, human embryonic stem cells, brain tissues, and neuronal cell lines, using either microarray or RNA sequencing (RNA-seq). These datasets had at least three replicates per group.

Five datasets reveal associations between expression levels of miRNAs and PD (Table 3), supporting their potential involvement in disease progression.

The microarray and RNA-seq datasets targeting long RNAs were mainly used for protein-coding sequences. A re-analysis of these datasets, starting with the re-annotation of microarray probes [201], and the re-alignment of sequencing reads [202] could provide a source of additional information on the regulation of known, and novel lncRNAs. Bioinformatics tools such as DCC [203] and findCirc [204] applied to RNA-seq datasets could be useful to discover novel circRNAs associated with PD.

## 10. Harmonization of High-Throughput Datasets and Clinical Data from Parkinson’s Disease Patients

There are several national and international efforts to harmonize clinical and multi-omics data for PD. The Michael J. Fox Foundation (MJFF) strives to make PD data available to the broader research community. Through their sponsored studies, they have collected or acquired clinical, omics, imaging, genomics, sensor, and patient-reported data. The largest projects are the Parkinson’s Progression Markers Initiative (PPMI, http://www.ppmi-info.org/), the MJFF investigation for the New Discovery of Biomarkers (BioFIND, https://biofind.loni.usc.edu/), and the MJFF LRRK2 Cohort Consortium. PPMI is a cohort of 423 de novo idiopathic PD patients, 196 healthy controls, 64 subjects with scans without evidence of dopaminergic deficit, 65 patients with PD risk factors of hyposomnia and sleep disorder, and 645 genetic cases (372 LRRK2, 246 GBA, 27 SNCA mutation carriers). PPMI omics data include genomics, transcriptomics, and DNA methylation data. The Luxembourgish National Centre for Excellence in Research on Parkinson’s Disease (NCER-PD) has been created to catalyze research aiming to improve the diagnosis and risk stratification of PD [12]. This may be achieved through the combination of accurate clinical and molecular data obtained from PD patients in order to develop novel biomarker signatures and refine the classification of subtypes of the disease. This is expected to allow an earlier and more specific diagnosis of PD to offer patients better and personalized treatments. A rich set of biospecimen, omics, and clinical data have been collected for 800 patients and 800 healthy controls. NCER-PD harmonizes clinical data captured as electronic case report forms (eCRFs) within a research electronic data capture (RedCAP) that makes it possible to exchange or combine clinical and omics data between PD datasets, such as the DeNoPa dataset [205].

## 11. Systems-Level Biomedical Data Mining Applied to the Discovery of RNA Biomarkers of Parkinson’s Disease

### 11.1. RNA-Based Computational Biomarker Discovery for PD

In recent years, due to the widespread use of high-throughput experimental molecular profiling approaches for the study of complex diseases, computational approaches for systems-level biomarker discovery have gained significant importance. Among the omics profiling approaches used in biomarker research, RNA transcriptomics profiling methods, such as RNA-seq or microarrays, provide some of the most popular and effective means for the systems-level study of disease-associated biospecimens, with high coverage of relevant biomolecules.

To explore these data sources effectively, available in silico approaches for biomarker discovery include both domain-agnostic feature selection and machine learning approaches to identify single-molecular markers or combinatorial biomarker signatures, and dedicated, domain-specific systems biology approaches for biomarker modeling. While generic machine learning methods are directly applicable to diverse types of input data, new cellular pathways and network-based biomarker discovery approaches often require more time-consuming, domain-specific data processing, but can provide biomarker models with improved accuracy, robustness and interpretability by exploiting prior biological domain knowledge. To illustrate the potential of these new system-level approaches, in the following chapter, current computational pathways and network analysis methods for RNA-based biomarker modeling are discussed, as well as the first representative applications in PD research.

### 11.2. Pathway- and Network-Based Biomarker Modeling for PD Research

Statistical and machine learning analyses of omics data often consider all measured biomolecules as independent variables or model their dependencies using correlations. However, RNAs, proteins, and metabolites have functional interrelations, which may only partly be reflected by correlations. Public pathway and molecular interaction databases provide a rich body of prior knowledge on direct physical and regulatory interactions between biomolecules, which can be exploited for systems-level omics analyses.

For this purpose, cellular pathways and networks can be represented at different levels of complexity. Univariate pathway analysis approaches disregard the network topology of cellular processes, and score their alterations using only the list of pathway members as input for over-representation statistics, such as Fisher’s exact test, or continuous tests, as implemented in the software tools GSEA [206] or PAGE [207]. More recently, multivariate pathway analysis methods have been proposed, which represent coordinated pathway alterations by aggregating measurement data for all pathway members via dimension reduction approaches into global pathway activity statistics, also called “pathway fingerprints” or “meta-genes”. These approaches can provide robust, pathway-level predictive features to fit machine learning models for diagnostic sample classification [208,209].

Since the publicly available pathway definitions do not capture all the experimentally verified molecular interactions in public interaction databases, unbiased network analyses may be applied as a further means to identify systems-level alterations in omics data. The corresponding algorithms investigate entire genome-scale molecular networks to identify sub-networks with coordinated activity changes. They provide more complete coverage of the cellular process alterations than pathway analysis methods, but often lack the interpretability of compact, manually curated pathway definitions [210,211]. Apart from identifying local network perturbations, some of the more recently developed algorithms also enable causal network explorations. This can, for example, be achieved by exploiting prior knowledge on directional regulatory or signaling relationships between biomolecules to infer the most probable upstream molecular network causes for observed downstream changes in omics data [212,213,214,215]. In particular, these methods help researchers to identify key transcription factors or regulatory RNAs whose disease-associated alterations may have important pathological or protective downstream effects.

One of the first examples for systems-level molecular pathway analysis of PD was presented in a study integrating transcriptomics and data from genome-wide association studies to determine consensus on disease-associated cellular processes, such as axonal guidance and focal adhesion pathways, through a statistical meta-analysis [216]. Later studies combined pathway enrichment analyses with topological network analysis to identify key miRNA networks altered in PD [217], integrated known PD-associated pathways within a molecular interaction map for visual omics data exploration [218] and applied graph-based network analyses, e.g., to identify shared molecular network alterations between PD and type 2 diabetes [219].

More recently, for the causal network analysis of PD, transcriptomics studies investigating inter-process correlations of individual pathways to determine likely upstream and downstream pathway alterations were presented [220]. Moreover, causal network reconstructions for PD were derived from co-expression analyses [221], and from exploiting prior biological knowledge from canonical networks [222]. These new causal analytical approaches have revealed robust upstream molecular network perturbations in PD, e.g., affecting RNA metabolism [220], with potential applications in biomarker research for early-stage PD.

While these initial representative studies highlight the potential of systems-level pathway/network analyses in PD research, the high heterogeneity of the PD molecular and clinical symptoms, and the limited sample sizes available in previous analyses still pose major challenges in the data analysis and modeling. Large-scale validation studies and meta-analyses across independent cohorts are therefore required in the future to substantiate the reported cellular processes alterations in PD and their utility for biomarker modeling.

## 12. Conclusions and Future Directions

Overall, the knowledge on a potential functional role and biomarker value of non-coding RNAs in PD development and progression is relatively scarce. This is especially true for circRNAs for which a deeper understanding of the functional role of their deregulation upon brain damage shall be acquired. In addition, although circRNAs are present in the blood—in leukocytes or encapsulated in extracellular vesicles–, whether they have some potential as PD biomarkers remains to be examined. Although some studies provide insights into the role of non-coding RNAs in the brain-heart axis, a better knowledge of the interactions between these two organs is required. This knowledge can be gained using a combination of high throughput data combined with machine learning analysis. Systems-level pathway and network analyses of transcriptomics data have the potential to reveal new biomarker signatures for PD diagnosis and prognosis. To favor the translation of initial transcriptomics-derived discoveries into clinically relevant diagnostic tests, more extensive collections of RNA profiling data for PD, covering different tissues and body fluids, are required. Cross-cohort analyses, integrating findings from multiple studies, will aid in identifying robust biomarkers. Ultimately, the identification of novel non-coding RNAs involved in PD progression may lead to the development of diagnostic tests or treatments for the patient’s benefit.

## Figures and Tables

**Table 1:** Regulation of miRNAs in body fluid samples from PD patients.

miRNA	Regulation	No. of Samples	Sample Type	Reference
miR-205 miR-24 *	↑ ↓	28 PD vs. 28 controls	CSF	[26]
miR-144-5p miR-200a-3p miR-542-5p	↑ ↑ ↑	44 PD vs. 42 controls	CSF	[27]
miR-153* miR-409-3p * miR-10a-5p let-7g3p miR-136-3p miR-433 * miR-30b miR-1 miR-19b-3p	↑ ↑ ↑ ↑ ↑ ↑ ↑ ↓ ↓	47 PD vs. 27 controls	CSF Exosomes	[28]
miR-4274	↓	28 PD vs. 6 controls	CSF	[29]
miR-626 *	↓	20 PD vs. 27 controls	CSF	[30]
miR-24 * miR-195 miR-19b	↑ ↑ ↓	109 PD vs. 40 controls	Serum Exosomes	[31]
miR-335-5p * miR-3613-3p miR-6865-3p	↑ ↑ ↑	16 PD vs. 8 controls	Serum	[32]
miR-150	↓	80 PD vs. 60 controls	Serum	[33]
miR-29c *	↑	51 PD vs. 20 controls	Serum	[34]
miR-146a miR-335-3 miR-335-5p *	↓ ↓ ↓	85 PD vs. 40 controls	Serum	[35]
miR-141 miR-214 miR-146b-5p miR-193a-3p	↓ ↓ ↓ ↓	169 PD vs. 180 Controls	Serum	[36]
miR-221	↓	138 PD vs. 112 controls	Serum	[37]
miR-29	↓	80 PD vs. 80 controls	Serum	[38]
miR-222 * miR-505 * miR-626 *	↓ ↓ ↓	42 PD vs. 30 controls	Plasma	[39]
miR-331-5p *	↑	31 PD vs. 25 controls	Plasma	[40]
miR-124 miR-137	↓ ↑	60 PD vs. 60 controls	Plasma	[41]
miR-30a-5p	↑	60 PD vs. 60 controls	Plasma	[42]
miR-105-5p	↑	319 PD vs. 273 controls	Plasma	[43]
miR-433 * miR-133b	↓ ↓	46 PD vs. 49 controls	Plasma	[44]
miR-27a Let-7a Let-7f miR-142-3p miR-222 *	↑ ↓ ↓ ↓ ↓	25 PD vs. 25 controls	Plasma	[45]
miR-331-5p miR-505 *	↑ ↑	52 PD vs. 48 controls	Plasma/exosomes	[46]
miR-132 *	↑	269 PD vs. 222 controls	Plasma	[47]
miR-7-5p miR-22-3p* miR-124-3p miR-136-3p * miR-139-5p miR-330-5p miR-433-3p miR-495-3p miR-132-3p miR-431-3p miR-128-3p miR-136-3p * miR-154-5p miR-323a-3p miR-338-3p miR-382-5p miR-409-3p * miR-410-3p miR-485-5p miR-22-3p *	↑ ↑ ↑ ↑ ↑ ↑ ↑ ↑ ↑ ↑ ↓ ↓ ↓ ↓ ↓ ↓ ↓ ↓ ↓ ↓	99 Idiopathic PD vs. 101 controls, 27 patients with GBA mutations vs. 101 controls and 26 SNCA-A53T mutation carriers vs. 101 controls	Plasma	[48]
miR-29c * miR-424 miR-30e-5p	↑ ↑ ↑	13 PD vs 10 non-PD	PBMC	[49]
miR-103a-3p miR-30b-5p miR-29a-3p	↑ ↑ ↑	46 PD vs. 46 controls	PBMC	[50]
miR-155-5p miR-146-5p	↑ ↓	37 PD vs. 43 controls	PBMC	[51]
miR-30c miR-26a	↓ ↓	19 PD vs. 13 controls	PBMC	[52]
miR-885 miR-17 miR-361	↑ ↑ ↓	36 PD vs. 16 controls	PBMC	[53]
miR-376a	↓	33 PD vs. 25 controls	PBMC	[54]
miR-7 miR-9-5p miR-9-3p miR-129 miR-132 *	↑ ↑ ↑ ↑ ↑	20 non-medicated PD vs. 18 medicated PD	Peripheral blood lymphocytes	[55]
miR-223 miR-153 *	↓ ↓	83 PD vs. 77 controls	Saliva	[56]
miR-874 miR-145-3p	↑ ↑	30 PD vs. 30 controls	Saliva	[57]

CSF: cerebrospinal fluid; PBMC: peripheral blood mononuclear cells; PD: Parkinson’s disease; vs.: versus; * miRNAs found to be differentially expressed in more than one cohort; ↑ or ↓ symbolize up or down regulation of miRNA expression.

**Table 2:** Regulation of lncRNAs in brain and blood samples of PD patients.

lncRNA	Regulation	No. of Samples	Sample Type	Reference
lincRNA-p21 MALAT1 SNHG1 NEAT1 * H19	↑ ↑ ↑ ↑ ↓	20 PD vs. 10 controls	Human brain specimens	[115]
AL049437 AK021630	↑ ↓	11 PD vs. 14 controls	Tissue samples	[118]
U1 RP11-462G22.1	↓ ↓	3 PD vs. 3 controls	Blood Leukocytes	[119]
AC131056.3-001 HOTAIRM1 lnc-MOK-6:1 RF01976.1-201	↑ ↑ ↑ ↑	72 PD vs. 22 controls	Blood Leukocytes	[120]
NEAT1 *	↑	61 PD vs. 42 controls	PBMC	[117]
AS-Uchl1	↓	68 PD vs. 65 controls	Plasma	[121]

PBMC: peripheral blood mononuclear cells; PD: Parkinson’s disease; vs.: versus; * lncRNAs found to be differentially expressed in more than one cohort; ↑ or ↓ symbolize up or down regulation of lncRNA expression.

**Table 3:** GEO datasets reporting associations between miRNAs and Parkinson’s disease.

GEO Reference	Profiling Technique	Main Observations	Sample Type	Reference
GSE72962	RNA-seq	125 miRNAs are regulated in PD A set of 29 miRNAs classifies PD	Frozen brain tissue from pre-frontal cortex of 29 PD patients and 33 controls.	[197]
GSE16658	Microarray	18 miRNAs are regulated in PD 11 miRNAs are over-represented in pathways linked to PD	PBMCs of 19 PD patients and 13 controls	[53]
GSE110719	RNA-seq	99 miRNAs are regulated in the substantia nigra of PD patients	Fibroblasts and iPSCs from 6 controls and nine PD Dopaminergic neurons from five controls and six PD.	[198]
GSE97285	RNA-seq	Several groups of miRNAs are regulated in PD at different disease progression stages	Brain samples from the amygdala—14 PD and 14 controls	[199]
GSE40915	RNA-seq	16 miRNAs are regulated in blood leukocytes of PD patient’s pre-treatment 11 miRNAs are regulated after brain stimulation, among which five vary inversely to disease evolution.	Blood leukocytes from nine PD and three controls.	[200]

## Abbreviations

PD	Parkinson’s disease
miRNA	MicroRNA
lncRNA	Long non-coding RNA
circRNA	Circular RNA
CSF	Cerebrospinal Fluid
PBMC	Peripheral blood mononuclear cells
PPMI	Parkinson’s Progressive Markers Initiative
NCER-PD	National Centre of Excellence in Research on Parkinson’s Disease
MPTP	1-methyl-4-phenyl-1,2,3,6-tetrahydropyridine
MPP	1-methyl-4-phenylpyridinium
LRRK2	Leucine-rich repeat kinase 2
HOTAIR	HOX transcript antisense RNA
NEAT1	Nuclear Paraspeckle Assembly Transcript 1
MALAT1	Metastasis Associated Lung Adenocarcinoma Transcript 1
BACE1-AS	Beta- Secretase 1 Antisense RNA
HAGLROS	HAGLR Opposite Strand LncRNA
*C. elegans*	*Caenorhabditis elegans*
GEO	Gene Expression Omnibus
